# Acute Sleep Curtailment Increases Sweet Taste Preference, Appetite and Food Intake in Healthy Young Adults: A Randomized Crossover Trial

**DOI:** 10.3390/bs10020047

**Published:** 2020-02-01

**Authors:** Eri Tajiri, Eiichi Yoshimura, Yoichi Hatamoto, Hideki Shiratsuchi, Shigeho Tanaka, Seiya Shimoda

**Affiliations:** 1 Graduate School of Environmental and Symbiotic Sciences, Prefectural University of Kumamoto, 3-1-100 Tsukide, Higashi-ku, Kumamoto 862-8502, Japan; et.nk02@gmail.com; 2 Faculty of Environmental and Symbiotic Sciences, Prefectural University of Kumamoto, 3-1-100 Tsukide, Higashi-ku, Kumamoto 862-8502, Japan; hideki-s@pu-kumamoto.ac.jp (H.S.); sshimoda@pu-kumamoto.ac.jp (S.S.); 3 Department of Nutrition and Metabolism, National Institute of Health and Nutrition, National Institutes of Biomedical Innovation, Health and Nutrition, Tokyo 162-8636, Japan; yhatamoto@nibiohn.go.jp (Y.H.); tanakas@nibiohn.go.jp (S.T.)

**Keywords:** sleep curtailment, sweet taste preference, active ghrelin, appetite, energy intake, *Ad libitum* meal

## Abstract

This study aimed to examine the effect of acute sleep curtailment on sweet taste preference, appetite and food intake, and the correlation between food intake and sweet taste preference or active ghrelin using a randomized crossover design (5 h sleep curtailment vs. 8 h control). Twenty-four participants (11 men) aged 21.4 ± 1.0 years, with BMI 19.8 ± 1.7 kg/m^2^, who habitually slept 5 h/night or more experienced interventions lasting three consecutive nights. Participants came into the laboratory for testing on day 4. Fasting blood tests were conducted at 8:00 a.m. to measure active ghrelin and leptin levels. Sweet taste preference was assessed by presenting five different concentration sucrose solutions at 9:00 a.m. *Ad libitum* intake at breakfast was assessed for 30 min from 9:30 a.m. Sweet taste preference was higher following sleep curtailment than control. Active ghrelin was likewise higher following sleep curtailment than control. Leptin did not differ between conditions. Energy intake was higher following sleep curtailment than control, being derived primarily from carbohydrates. However, sweet taste preference and active ghrelin did not correlate with energy intake. These results suggest that acute consecutive sleep curtailment increases sweet taste preference, active ghrelin, and energy intake in healthy young adults.

## 1. Introduction

Epidemiologic studies have concluded that sleep curtailment contributes to obesity [1,2]. A meta-analysis of the effect of sleep curtailment on energy balance reported that energy intake was increased by 385 kcal in the sleep curtailment condition compared with normal sleep [3]. Although numerous studies have investigated the association between sleep curtailment and energy intake [4,5,6,7,8], the factors that increase energy intake in the sleep curtailment condition are not understood.

Several studies have reported that energy intake, especially from fat and carbohydrate, increases in the sleep curtailment condition compared with a control sleep condition. One fMRI study reported that neural regions involved in pleasure-seeking and food-related behaviors were activated by unhealthy compared with healthy foods in the sleep curtailment condition [9]. A recent systematic review suggested that sweet taste preference, but not the threshold, is associated with energy intake [10]. Additionally, it has been reported that sweet taste preference increases in the sleep curtailment condition (≤7 h) and in the control condition (>7 h) [11]. Therefore, the increase in sweet taste preference caused by lack of sleep might lead to the selection of high-calorie and unhealthy foods. However, to the best of our knowledge, there has been only one study that has examined the effect of sleep curtailment on sweet taste preference [11]. It is therefore necessary to clarify the factors by which sleep curtailment increases energy intake.

In a previous cross-sectional study, sleep loss was associated with increase in ghrelin level [12]. On the other hand, meta-analysis of intervention studies has reported that sleep loss was not associated with ghrelin level [13]. Recently, a number of studies have explored the association between food intake and the orexigenic hormone ghrelin during sleep curtailment [4,14,15,16,17]. Broussard et al. [8] reported that energy intake from sweet snacks and ghrelin levels during sleep curtailment increased compared with control sleep. Furthermore, this study found a significant correlation between energy intake from sweet snacks and ghrelin level. However, these findings are not consistent with the results from several other studies [4,14,15,16,17]. In addition, correlations of appetite-regulating hormones and sweet taste preference with food intake deserve further study.

The present study aimed to investigate the effect of sleep curtailment on sweet taste preference and appetite-regulating hormones, and the association between these factors and food intake. We hypothesized that sleep curtailment would increase food intake by changing sweet taste preference and levels of the orexigenic hormone ghrelin.

## 2. Materials and Methods

### 2.1. Participants

Participants were 24 young healthy adults (13 women and 11 men). Inclusion criteria were as follows: 1) ≥20 years old; 2) body mass index (BMI) < 25 kg/m^2^; 3) habitual sleep duration ≥ 5 h, assessed by the Pittsburgh Sleep Quality Index (PSQI) [4]; 4) no use of medication and free of diseases such as diabetes, hypertension or congenital disorders; 5) nonsmoking; 6) no problems sleeping, corresponding to a PSQI score <10; 7) chronotype classified as definitely not an evening type, based on a score >42 on the Japanese version of the morningness–eveningness questionnaire.

### 2.2. Study Design and Procedure

**Figure 1:**
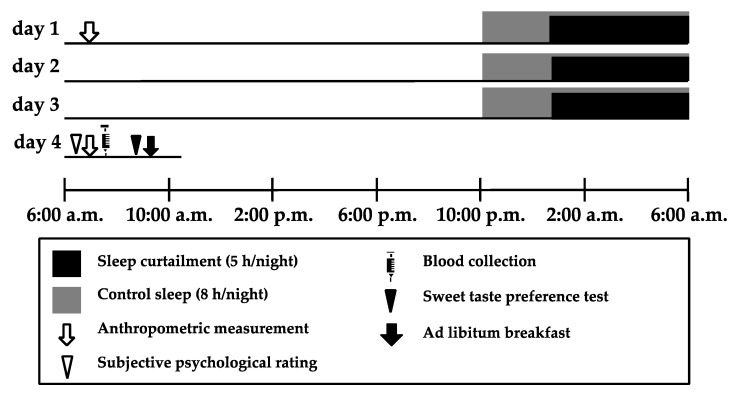
Experimental protocol. The protocol used a randomized crossover design with a sleep curtailment condition (5 h/night) and a control sleep condition (8 h/night). Both conditions were across 3 consecutive nights. The washout period between conditions was at least 3 weeks to minimize crossover effects. Participants were asked to come to the laboratory at 8:00 a.m. after fasting since getting up on day 1. We then collected anthropometric data from the participants, informed them about the study procedure, and asked them to answer a questionnaire assessing their habitual sleep patterns, caffeine and alcohol intake, and chronotype. Participants were free to carry out daily activities as normal and sleep at home from day 1 to 3. During the intervention periods, participants decided on their wake-up time to arrive on time at the laboratory at 8:00 a.m. on day 4. On day 4, participants were asked to come to the laboratory at 8:00 a.m., where we collected anthropometric data from them, asked them to perform subjective ratings, and collected blood samples. The sweet taste preference test was conducted at 9:00 a.m., and the *Ad libitum* breakfast began at 9:30 a.m.

This study used a randomized crossover design with a sleep curtailment condition (5 h/night) and a control sleep condition (8 h/night). The procedure for the study is shown in Figure 1. Participants were informed of the order in which they would experience the conditions on the first day of the study. Both conditions lasted for 3 consecutive nights. The washout period between conditions was at least 3 weeks to minimize crossover effects. Women took part in the study within 2 weeks after their menstrual period because appetite and sleepiness are affected by the menstrual cycle [18,19]. Participants were asked to go to bed at the same time as usual on prior day of entering protocol. Participants were free to engage in daily activities as normal, with the exception of sleeping. They were asked to sleep at home from day 1 to day 3, with the wake-up time during the intervention period chosen by participants as being suitable for coming to the laboratory at 8:00 a.m. on day 4. Participants were instructed not to consume drinks containing caffeine or alcohol during the intervention period and not to sleep except during the prescribed sleep period. 

On day 1, participants were asked to come to the laboratory at 8:00 a.m., having fasted after getting up. We then collected anthropometric data from the participants, instructed them about the study procedure and asked them to answer questionnaires assessing their habitual sleep patterns, caffeine and alcohol intake, and chronotype. Participants were fitted with two accelerometers to evaluate their physical activity and sleep from 8:00 a.m. on day 1 to 8:00 a.m. on day 4, except while bathing. Participants were asked to go to bed and turn off the lights for a prescribed duration of sleep from day 1 to day 3. On day 4, participants were asked to come to the laboratory at 8:00 a.m. We then collected anthropometric data, assessed their subjective psychological rating, and collected a blood sample from each participant. The sweet taste preference test was conducted at 9:00 a.m., and participants ate breakfast *Ad libitum* from 9:30 a.m. onwards. The protocol was registered as a randomized controlled trial entitled “The effect of sleep curtailment on appetite and food preference” under the University Hospital Medical Information Network (UMIN) registration number UMIN000028906 and approved by the ethical committee of the Prefectural University of Kumamoto (28–22). All participants gave written informed consent to the purpose, methods, and significance of the study.

### 2.3. Sleep Assessment

Habitual sleep time and sleep quality were assessed using the PSQI. Participants recorded the time at which they went to bed, their wake-up time, nap times, and sleep satisfaction within 10 min of getting up from day 2 to 4 in subjective sleep records. We asked them to record nap times if they involuntarily took a nap that might be possible to occur except while the prescribed sleep period. Sleep satisfaction was assessed using a four-point scale (1 to 4), with the question “How satisfied are you with your sleep last night?” rated as: (1) dissatisfied; (2) a little dissatisfied; (3) a little satisfied; (4) satisfied. Sleep was assessed objectively using a MicroTag accelerometer (MTN-220; ACOS, Nagano, Japan) [20]. Sleep analysis was conducted using SleepSign Act 2.0 (KISSEI COMTEC, Nagano, Japan). Participants were asked to wear the MicroTag accelerometer from 8:00 on day 1 to 8:00 a.m. on day 4, except while bathing.

### 2.4. Anthropometric Measurements

Anthropometric measurements were conducted at 8:00 a.m. on day 1 and 4, with participants dressed in light clothing, without shoes and socks. Height and weight were measured using a stadiometer with a weight scale (DC-250; Tanita, Tokyo, Japan). BMI was calculated in units of kg/m^2^.

### 2.5. Subjective Psychological Rating

At 8:00 a.m. on day 4, six variables (hunger, appetite, desire for sweet foods, desire for fatty foods, sleepiness, and fatigue) were assessed using subjective psychological ratings using a 0.0–10.0 cm visual analog scale (VAS). The six variables were assessed as described in an earlier study [21]. The VAS was evaluated in units of 0.1 cm. For hunger, the question “How hungry do you feel right now?” was anchored by “not at all hungry” on the left and “extremely hungry” on the right. For appetite, namely the desire for sweet foods and the desire for fatty foods, the questions “How strong is your desire to eat right now?”, “How strong is your desire to eat sweet foods right now?”, and “How strong is your desire to eat fatty foods right now?” were anchored by “not at all” and “extremely”. For sleepiness, “How sleepy do you feel right now?” was anchored by “not at all sleepy” and “extremely sleepy”; and for fatigue, “How fatigued do you feel right now?” was anchored by “not at all” and “feeling so fatigued that I can’t move”.

### 2.6. Blood Collection

Blood samples were obtained from the antecubital vein on the morning of day 4 at 8:00 a.m. after participants had been fasting since the last meal of the previous day. Serum biochemistry analysis was conducted by LSI Medience Co. (Tokyo, Japan). Biochemical parameters included active ghrelin (Active Ghrelin ELISA kit; SCETI K.K, Tokyo, Japan) and leptin (Radioimmunoassay; Merck Millipore, MA, USA). The coefficient of variation between samples was within 10%. 

### 2.7. Sweet Taste Preference Test

The sweet taste preference test was conducted at 9:00 a.m. on day 4. The temperature of the experimental room was set to 25 °C, and the door was kept closed. The test was conducted according to the method of Asao, Luo, and Herman [22]. The concentrations of sucrose solutions used were 3%, 6%, 12%, 24%, and 36% w/v. Sucrose solutions were prepared from food grade sucrose (FUJIFILM Wako Pure Chemical Corporation, Osaka, Japan) and distilled drinking water (FUJIFILM Wako Pure Chemical Corporation, Osaka, Japan). At first, participants rinsed their mouth with distilled water and spat it out. Then, a pair of disposable medicine cups labeled “A” and “B” containing 5 mL each of different concentration sucrose solutions were presented to the participant. The first pair of cups “A” and “B” presented contained sucrose solutions from the middle of the concentration range (6% and 24% w/v). Participants tasted each solution for 5 s and then spat it out. They next drew a circle around the letter “A” or “B” on the cups to indicate which solution they liked better and returned them. We did not inform participants about how the solutions differed. If the participant liked the higher concentration, the next pair of solutions presented were the selected concentration and the solution with one step higher concentration. If the participant liked the lower concentration, the next pair of solutions presented was the selected concentration and the solution with one step lower concentration. The inter-pair intervals were set at 30–60 s. The series of presentations was complete when the participant chose one concentration two times in a row; the concentration chosen was recorded as the preferred concentration of the participant. Each pair of solutions was presented in a randomized order. The solution assigned to cup “A” was tasted first, and the one assigned to cup “B” was tasted second. The solutions contained in cups “A” and “B” were assigned at random.

### 2.8. Ad libitum Breakfast

At 9:30 a.m. on day 4, *Ad libitum* food intake was assessed. The temperature in the experimental room was set to 25 °C, and the door was closed. In total, 32 types of food were presented. *Ad libitum* foods comprised: staple foods (rice, granola, rice casserole, pizza, two kinds of spaghetti, and croissants); main dishes (takoyaki with sauce and mayonnaise, grilled salmon, fried fish, omelette, beef croquette, fried chicken, and fried spring rolls); side dishes (six kinds of daily dishes and three kinds of salads); drinks (milk, and orange juice); and desserts (yogurt, chocolate, vanilla ice cream, chocolate ice cream, and cheesecake). The nutritional component of *Ad libitum* meals is shown in supplemental table. Participants selected and ate foods alone from 9:30 a.m. onwards. They were instructed to finish eating within 30 min. They were asked to eat as they wished and to continue selecting foods to eat until they were full. The researchers confirmed that no food was left in the dish after participants finished eating. The quantity of foods selected by participants was recorded by the researchers. All interventions were carried out in an identical fashion regarding food portion sizes, foods provided, food layout and the setup of the experimental room.

### 2.9. Statistical Analysis

All data are presented as the mean ± standard deviation. Sleep records were assessed to calculate the mean across 3 nights. Habitual caffeine and alcohol intake before the intervention period were calculated as the intake of each per week. Comparisons between the sleep curtailment condition and the control sleep condition were conducted using a mixed-model ANOVA. The results for blood biochemistry and food intake adjusted for sex and the results for blood biochemistry adjusted for the time of the last meal were assessed using a mixed-model ANOVA. Correlations between food intake, active ghrelin level, and sweet taste preference and between sweet taste preference and subjective desire to sweet were analyzed using the Pearson correlation coefficient. Active ghrelin and the time of last meal from 23 participants were analyzed, because one participant had missing data. The significance level for statistical tests was set at 0.05. All statistical analyses were conducted using IBM SPSS 22.0 (SPSS Inc., Chicago, IL, USA).

## 3. Results

### 3.1. Participant Characteristics

Table 1 shows the characteristics of the participants before the intervention. No participants had a chronotype of definitely morning type or definitely evening type. The time of the intervention in women was 6.0 ± 3.3 d after menstruation and did not differ between conditions (*p* > 0.05). Habitual sleep duration did not differ between men and women (*p* > 0.05).

### 3.2. Body Composition and Sleep Characteristics

Body weight on day 1 and 4 did not differ between participants in the sleep curtailment condition and those in the control sleep condition (day 1: 55.7 ± 7.2 vs. 55.9 ± 7.1 kg; F = 0.881, *p* = 0.358; day 4: 56.3 ± 7.4 vs. 56.3 ± 7.0 kg, F < 0.001, *p* = 0.988). The change in weight from day 1 to 4 was not caused by a condition × day interaction (F = 0.747, *p* = 0.396). Body weight on day 4 significantly increased compared with that on day 1 for both conditions (both *p* < 0.05). 

Table 2 shows the objective characteristics of sleep in participants during the intervention. The self-reported bed time and wake time were significantly different between conditions (bed time; 1:32 a.m. ± 0:32 vs. 10:42 p.m. ± 0:35, F = 96932.473, *p* < 0.001, getting-up time; 6:56 a.m. ± 0:31 vs. 6:56 a.m. ± 0:36, F = 59311.426, *p* < 0.001). Subjective sleep satisfaction for those in the sleep curtailment condition was significantly lower than for those in the control sleep condition (2.1 ± 0.6 vs. 3.0 ± 0.7; F = 41.537, *p* < 0.001). The self-reported nap time did not differ between conditions (10 ± 13 vs. 5 ± 8 min; F = 2.363, *p* = 0.138).

### 3.3. Subjective Psychological Rating and Blood Biochemistry

The results of the subjective psychological rating are shown in Table 3. Subjective sleepiness and fatigue in the sleep curtailment condition were significantly higher than those in the control sleep condition. However, subjective hunger, appetite, desire for sweet foods, and desire for fatty foods did not differ between conditions. 

Table 3 shows the results of active ghrelin and leptin level measurements. The mean fasting duration from the time of the last meal on the previous day to the time of blood sample collection differed significantly between conditions (sleep curtailment condition vs. control sleep condition: 9:47 ± 1:58 vs. 12:01 ± 1:17 h; F = 28.386, *p* < 0.001). The duration of fasting ranged from 6:40 a.m. to 1:20 p.m. in the sleep curtailment condition and from 10:00 a.m. to 2:30 p.m. in the control sleep condition. Active ghrelin and leptin levels in women were significantly higher than those in men. The active ghrelin level in the sleep curtailment condition was significantly higher than that in the control sleep condition (*p* = 0.035). However, leptin level did not significantly differ between conditions. After adjusting for sex, the results for active ghrelin and leptin levels remained the same. After adjusting for the time of the last meal, active ghrelin and leptin levels did not significantly differ between conditions (active ghrelin: F = 3.152, *p* = 0.089; leptin: F = 0.799, *p* = 0.381).

### 3.4. Sweet Taste Preference 

**Figure 2:**
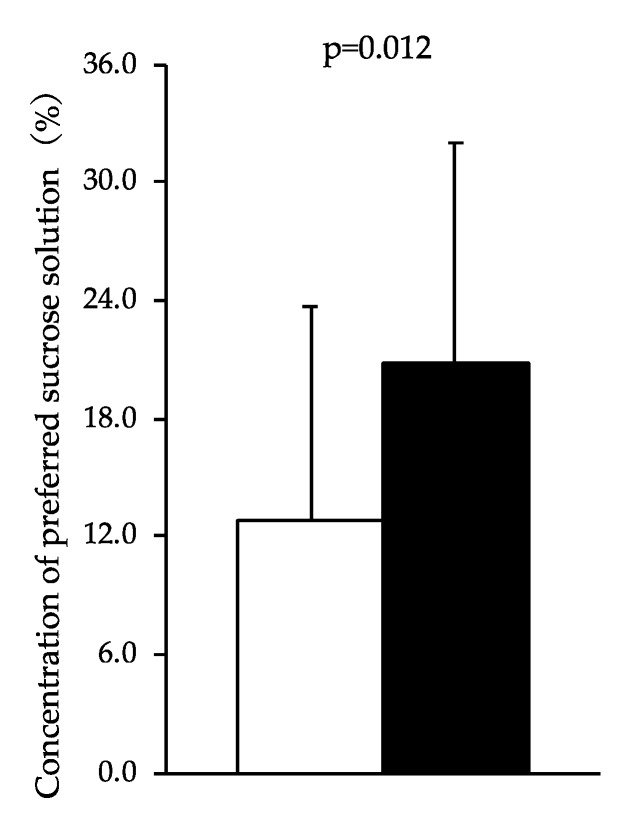
Concentration of preferred sucrose solution chosen by participants after the sleep curtailment condition and control sleep condition. Black bars represent the sleep curtailment condition and white bars represent the control sleep condition. The black and white bars and error bars show the mean and standard deviation, respectively.

Figure 2 shows the preferred concentration of sucrose solution chosen in each condition. The preferred concentration of sucrose solution in the sleep curtailment condition was significantly higher than that in the control sleep condition (20.9 ± 11.1 vs. 12.9 ± 10.8 %; F = 7.388, *p* = 0.012). The change in sweet taste preference was not correlated with the change in subjective desire for sweet.

### 3.5. Food Intake during the Ad libitum Breakfast

Table 3 shows food intake by participants during the *Ad libitum* breakfast. Energy and carbohydrate intakes in those in the sleep curtailment condition were significantly higher than those in the control sleep condition. However, protein and fat intakes did not differ between conditions. The weights of the food and the macronutrients consumed (protein, fat, and carbohydrate) were both significantly higher in the sleep curtailment condition than those in the control sleep condition. However, the energy density did not differ between conditions. After adjusting for sex, the results for energy and macronutrients intake remained the same.

Using correlation analysis, the change in energy intake was not correlated with the change in active ghrelin level (r = 0.219, *p* = 0.314). The correlation between the change in energy intake and the change in sweet taste preference was not significant (r = 0.228, *p* = 0.285). The change in macronutrients intakes (protein, fat, and carbohydrate) was not correlated with the change in sweet taste preference.

## 4. Discussion

The present study focused on the effect of sleep curtailment on sweet taste preference and appetite and the association between these variables and food intake. Sweet taste preference, active ghrelin level, and energy and carbohydrate intakes were significantly higher in the sleep curtailment condition than in the control sleep condition. Contrary to our hypothesis, we could not find significant correlations between food intake and either active ghrelin level or sweet taste preference. 

A previous study reported that the increase in energy intake related to sleep curtailment is associated with nonhomeostasis [23]. Our study assessed sweet taste preference as one potential nonhomeostatic response. The strength of our study is that we assessed the effect of sleep curtailment on both sweet taste preference and energy intake. We expected that the change in taste preference caused by sleep curtailment might induce an increase in food intake. However, we found that sweet taste preference was not correlated with energy intake, although three nights of 5 h of sleep curtailment increase sweet taste preference and the intake of energy and carbohydrates. In contrast to these results, a prior study reported that sweet taste preference and energy intake did not differ between a single night’s <7 h of sleep curtailment and ≥7 h of control sleep and that sweet taste preference was associated with energy intake after sleep curtailment [11]. This difference likely resulted from differences in the intervention conditions. The strong sleep intervention in our study might have caused a greater increase in sweet taste preference than that observed in the prior study. We did not find a direct correlation between sweet taste preference and energy intake. However, our findings showed that sweet taste preference was increased as a result of sleep curtailment to the same extent as participants in this group choosing to add about one and half tablespoons (16 g) of sugar in 200 mL of coffee. Interestingly, subjective preference for sweets was not different between conditions. The discrepancy of this results was unclear, and it needs to be considered by further studies. 

A prior study conducted in only male participants reported that the total ghrelin level in the sleep curtailment group was higher than that in the control sleep group [24]. However, other studies have reported no significant difference in the total ghrelin level between conditions [4,14,16,17]. Total ghrelin includes active ghrelin and desacyl-ghrelin, with active ghrelin leading to food intake [25]. In our study, the active ghrelin level in the sleep curtailment condition was significantly higher than in the control sleep condition. However, few studies to date have reported data regarding the active ghrelin level during sleep curtailment. In one previous study, the active ghrelin level was found not to differ between the sleep curtailment and control sleep groups in either men or women [24]. The analysis adjusted time from last meal did not give the difference of active ghrelin level. Our result is consistent with that earlier result that active ghrelin level was not different between conditions. However, active ghrelin levels might be indirectly influenced by sleep curtailment through delaying meal timing.

In the present study, changes in energy intake and carbohydrate intake were not correlated with the change in active ghrelin level measured in the morning. Broussard et al. [8] found that the increase in total ghrelin in men measured in the evening during sleep curtailment was significantly correlated with a higher consumption of calories from sweet foods (r = 0.48). Furthermore, active and total ghrelin levels were higher in the evening than in the morning [8,26]. Therefore, the inconsistencies between these findings and ours might be because of methodological differences in the timings of blood sampling and meals.

In our previous study, we did not observe an increase in energy intake during the *Ad libitum* breakfast because we only offered a few kinds of food [27]. Therefore, to improve on this in the present study, we provided 32 kinds of foods at the *Ad libitum* breakfast. As a result, energy intake and carbohydrate intake in the sleep curtailment condition were higher than those in the control sleep condition. The results of the present study are consistent with a meta-analysis indicating that energy intake increases by 385 kcal after sleep curtailment [3]. In addition, our study found that carbohydrate intake contributes to energy intake after sleep curtailment. However, contrary to our hypothesis, energy intake was not correlated with sweet taste preference and active ghrelin level. McNeil and St-Onge [28] suggested that the effect of sleep curtailment on energy intake exhibits large differences between individuals. In our study, the range of individual differences in energy intake between conditions was -261 to 679 kcal. We found both participants who increased their energy intake and participants who did not change their intake or decreased it. To clarify factors of interindividual differences in energy intake, further studies with greater numbers of participants would be required. Furthermore, a previous study reported that the endocannabinoid 2-arachidonoylglycerol is an index of the activation of the brain reward system increased after sleep curtailment [29]. Therefore, it is necessary to investigate other factors that might enhance energy intake after sleep curtailment. 

Our study has some limitations. First, we did not assess the effect of chronic sleep curtailment and sleep curtailment by early awakening. The effects of sleep curtailment with a long period or advanced wake time would be expected to differ from acute effects of sleep curtailment with delayed bed time. Previous study has reported that chronic sleep restriction does not alter subjective hunger levels and appetite hormones, but rather, they are more circadian-rhythm-dependent [30]. Olfactory responses seem to change based on circadian timing [31], which could also impact taste response. Additionally, Herz et al. reported that olfactory sensitivity was influenced by circadian timing [31]. Therefore, circadian timing might also impact taste response. Additional studies need to focus not only on sleep condition but also on circadian rhythm. Second, the food intake and timings of meals on day 3 were not controlled, so we cannot exclude the possibility that these meals affected levels of appetite-regulating hormones. Although we calculated our statistical results, including adjustments for the time of the last meal, future studies should examine how the results differ if the dietary composition and meal time on the day prior to blood sampling are controlled. Third, the effect of sleep curtailment on energy intake during a whole day or during snacking were not revealed. A number of previous studies have reported that sleep curtailment increases energy intake during late night meals and snacks [4,5,7,8]. If a future study were to examine the total energy intake or intake of snacks, it might be possible to show a significant association between sweet taste preference and energy intake. There were two participants in the sleep curtailment condition and a participant in the control sleep condition who napped for an hour. A participant napped for 1 h 20 min in the sleep curtailment condition. In the analysis, after excluding those who had napped, the results in this study were not different.

## 5. Conclusions

The present study found that acute consecutive sleep curtailment increased sweet taste preference, active ghrelin level, and energy and carbohydrate intakes at an *Ad libitum* breakfast. However, the present study did not find a correlation between energy intake and sweet taste preference or active ghrelin. Further studies should examine individual and sex differences in the effect of sleep curtailment and how sleep curtailment affects food intake during the whole day, late at night, during snacking, and the release of food reward signals, such as 2-arachidonoylglycerol. Furthermore, future studies also need to investigate the effect of chronic sleep curtailment on food intake, sweet taste preference, and appetite.

## Figures and Tables

**Table 1:** Characteristics of the participants before the intervention.

	All (n = 24)			Men (n = 11)			Women (n = 13)		
Age (years)	21.4	±	1.0	21.5	±	0.8	21.3	±	0.8
Body weight (kg)	55.6	±	7.2	61.0	±	5.6	51.0	±	5.0
BMI (kg/m^2^)	19.8	±	1.7	20.5	±	1.9	19.2	±	1.1
PSQI Score	4.9	±	1.8	5.5	±	1.4	4.4	±	1.9
Bed time	12:37 a.m.	±	0:47	12:51 a.m.	±	0:48	12:25 a.m.	±	0:43
Wake-up time	7:41 a.m.	±	0:44	8:05 a.m.	±	0:42	7:20 a.m.	±	0:35
Time in bed (min/night)	423	±	38	433	±	33	415	±	40
Total sleep time (min/night)	408	±	49	409	±	52	407	±	49
Sleep efficiency (%)	96.5	±	9.2	94.4	±	10.3	98.2	±	8.3
Habitual caffeine intake (mg/day)	159	±	105	184	±	100	137	±	109
Habitual alcohol intake (mg/day)	10	±	26	20	±	36	1	±	1
Chronotype (n, %)									
Moderate morning type	2	,	8.3	1	,	9.1	1	,	7.7
Intermediate type or neither type	19	,	79.2	8	,	72.7	11	,	84.6
Moderate evening type	3	,	12.5	2	,	18.2	1	,	7.7
Definite evening type or definite morning type	0	,	0	0	,	0	0	,	0

Age, body weight, BMI, PSQI (Pittsburg Sleep Quality Index), and habitual caffeine and alcohol intake are shown as the mean ± standard deviation. Chronotype is shown as the number of participants and the percent relative to the total number of participants.

**Table 2:** Assessment of the objective sleep characteristics of participants across three nights in the sleep curtailment and control sleep conditions.

	Sleep Curtailment Condition			Control Sleep Condition			F	P
Bed time	1:32 a.m.	±	0:31	10:43 p.m.	±	0:38	1566.676	<0.001
Sleep-onset time	1:43 a.m.	±	0:31	10:58 p.m.	±	0:38	751.825	<0.001
Awakening time	6:48 a.m.	±	0:35	6:48 a.m.	±	0:40	0.057	0.814
Getting-up time	6:54 a.m.	±	0:34	6:56 a.m.	±	0:40	0.208	0.653
Sleep period time (min/day)	304	±	20	471	±	22	1030.272	<0.001
Time in bed (min/day)	321	±	16	493	±	19	2251.343	<0.001

Values represent the mean ± standard deviation (mean of three nights). Sleep period time means duration from sleep-onset time to awakening time. Time in bed means duration from bed time to getting-up time.

**Table 3:** Subjective psychological ratings, active ghrelin level, leptin level, and *Ad libitum* food intake at breakfast of participants in the sleep curtailment and control sleep conditions.

	Sleep Curtailment Condition			Control Sleep Condition				
	Ave	±	SD	Ave	±	SD	F	P
Subjective psychological ratings								
Hungry (cm)	6.0	±	2.6	5.4	±	2.3	3.284	0.084
Appetite (cm)	5.4	±	2.6	5.4	±	1.8	0.001	0.976
Desire to sweets (cm)	4.2	±	2.2	4.4	±	2.4	0.482	0.495
Desire to fatty foods (cm)	3.5	±	2.6	2.7	±	2.4	2.129	0.159
Sleepiness (cm)	6.4	±	2.1	3.0	±	2.4	47.015	<0.001
Fatigue (cm)	5.6	±	2.1	2.8	±	2.2	38.574	<0.001
Blood biochemistry								
Active ghrelin (fmol/ml)*	22.8	±	10.6	20.1	±	8.7	5.020	0.035
Leptin (ng/ml)	17.4	±	10.6	17.4	±	10.9	0.005	0.946
*Ad libitum* food intake								
Energy intake (kcal)	788	±	384	674	±	281	6.547	0.018
Protein (kcal)	88	±	52	76	±	40	3.081	0.093
Fat (kcal)	308	±	173	272	±	149	3.287	0.083
Carbohydrate (kcal)	397	±	187	330	±	120	8.109	0.009
P (%/Energy)	10.9	±	2.1	11.1	±	2.2	0.183	0.673
F (%/Energy)	38.4	±	6.9	39.7	±	7.8	0.846	0.367
C (%/Energy)	51.5	±	7.7	49.7	±	8.0	1.210	0.283
Weight of food intake (g)	573	±	256	492	±	193	4.674	0.041
Weight of macronutrient intake (g)	155	±	74	132	±	52	7.189	0.013
Energy density (kcal/g)	1.4	±	0.3	1.4	±	0.3	0.058	0.812

Values represent the mean ± standard deviation. The weight of macronutrient intake was calculated by adding the weight of protein, fat, and carbohydrate together. Energy density was calculated as the energy intake (kcal)/weight of food intake (g). *; n=23.

## Supplementary Materials

The following are available online at https://www.mdpi.com/2076-328X/10/2/47/s1, Table S1: nutritional component of *Ad libitum* meals.
